# Force Relaxation Characteristics of Medium Force Orthodontic Latex Elastics: A Pilot Study

**DOI:** 10.5402/2011/536089

**Published:** 2011-07-06

**Authors:** Daniel J. Fernandes, Gisele M. Abrahão, Carlos N. Elias, Alvaro M. Mendes

**Affiliations:** ^1^Department of Orthodontics, State University of Rio de Janeiro, 20551-030 Rio de Janeiro, Brazil; ^2^Department of Material Sciences, Military Institute of Engineering, 22290-270 Rio de Janeiro, Brazil

## Abstract

To evaluate force extension relaxation of different brands and diameters of latex elastics subjected to static tensile testing under an apparatus designed to simulate oral environments, sample sizes of 5 elastics from American Orthodontics (AO), Tp, and Morelli Orthodontics (Mo) of equivalent medium force, (3/16, 1/4, and 5/16 inch size) were tested. The forces were read after 1-, 3-, 6-, 12- and 24-hour periods in Emic testing machine with 30 mm/min cross-head speed and load cell of 20 N. Two-way ANOVA and Bonferroni tests were used to identify statistical significance. There were statistically differences among different manufacturers at all observation intervals (*P* < 0.0001). The relationships among loads at 24-hour time period were as follows: Morelli>AO>Tp for 3/16, 1/4, and 5/16 elastics. The force decay pattern showed a notable drop-off of forces until 3 hours, a slight increase in some groups from 3–6 hours and a more homogeneous force pattern over 6–24 hours.

## 1. Introduction

Elastics made from natural rubber supplying orthodontic force continue to be in common use, mainly because of their favorable characteristics: low cost and high flexibility with greater ability to return to their original dimensions after substantial deformation [1]. It is easy for patients to change the elastics by themselves and maintain good oral hygiene. Elastics made from natural rubber were first introduced by Baker and have been applied up to the present [2].

“Elastomer” is the general term given to synthetic polymer materials. Natural rubber is also an elastomer, but not all elastomers can be called “rubber”. 

The original latex is the natural sap tapped directly from the rubber tree. It contains 25% to 40% of rubber hydrocarbon (*cis*-1, 4 polyisoprene) with small amounts of protein material and fatty acids [3]. The elastic properties of such materials depend on irregular twisted arrangements of very long molecular chains linked together at certain points by covalent bonds between different atoms such as sulphur with 2 carbon atoms. All elastomeric materials, including those made from latex (natural) rubber, undergo fatigue and creep force relaxation, which results in force decay that is likely to be accentuated under adverse environmental conditions, including those associated with the oral cavity. It has been a common finding that rubber elastics in a watery or oral environment lose between 10% and 40% of their initial force between 30 minutes and 24 hours after they are applied [3].

The force provided by elastics is directly related to the amount of extension between the 2 attachment points. The distance between these points varies from patient to patient, depending on the particular orthodontic purpose such as intermaxillary or intramaxillary tension, and on the expected amounts of jaw opening and closing in the case of intermaxillary elastics [3]. Liu et al. proposed that the normal range of clinical use during talking and chewing is between 20 and 50 mm [4]. 

Latex has been extensively used in orthodontics since the advent of the specialty. Latex elastics are used for final detailing of the occlusion and fixation of the maxilla and mandible together after surgery. Mechanotherapy in orthodontics often involves the use of interarch latex elastics to correct sagittal discrepancies or vertical elastics to improve the interdigitation of teeth. Whereas these auxiliaries are replaced on a daily basis, a concern associated with their use pertains to the force relaxation of the materials [4]. The orthodontist must be able to choose an elastic band with force-extension characteristics that are most suitable for the particular tooth movement required. This means that the orthodontist must know the force-extension characteristics of the range of elastics at his/her disposal [3].

There have been few reports about the properties or uses of orthodontic “rubber bands” as distinct from synthetic elastomers during the last 20 years, perhaps because orthodontists have been familiar with their use for many more than 20 years. In recent years, more interest in elastic materials has focused on the properties of the synthetic elastomers that have been developed for orthodontic use such as elastic threads, ligating modules, and elastic chains [3]. 

This pilot study was implemented to evaluate the characteristics of force degradation when the testing was conducted at different times during a 24-hour period. We have attempted to collect experimental information that will provide guidelines for in vitro mechanical analysis of latex elastics.

## 2. Materials and Methods

Five medium force orthodontic latex elastics were investigated at a 3/16-inch, 1/4-inch, and 5/16-inch size from American Orthodontics (AO), Tp, and Morelli Orthodontics. Force measurements were made at 6 time intervals: 0, 1, 3, 6, 12, and 24 hours making a total of 270 samples. They were within their expiration dates and stored in sealed plastic packages in a cool and dark environment.

**Figure 1:**
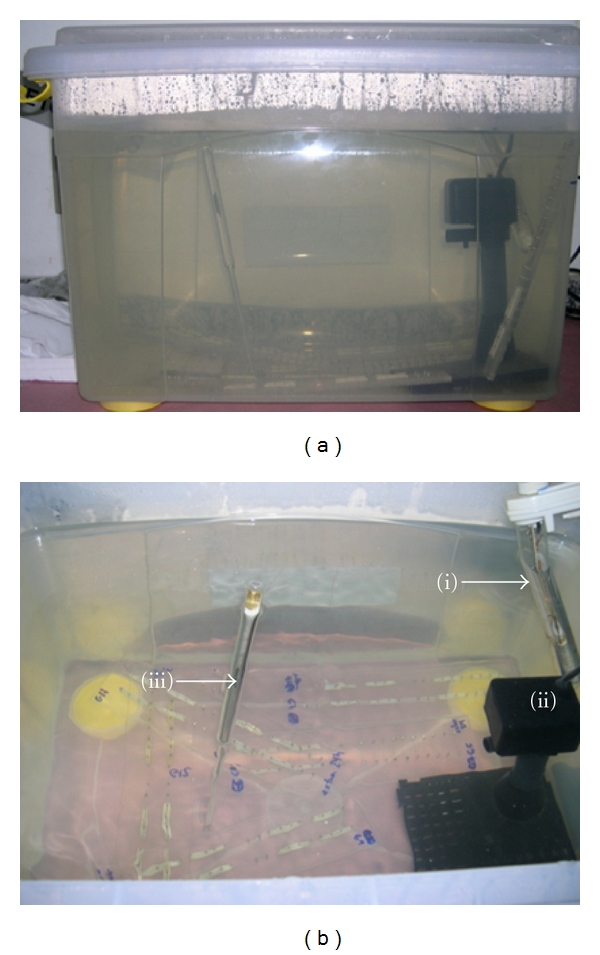
Special apparatus to simulate oral environments of temperature and humidity: (a) water tank, (b) view of tank apparatus: (i) water heater, (ii) water bomb, and (iii) thermometer.

A special apparatus was designed to simulate oral environments of temperature and humidity (Figure 1). The apparatus consisted of a plastic tank with 15 gallons of deionized water maintained at 37°C by a submersible water heater with accuracy of ±0.5°C (Magic Heater, 7 Stars Co, China) and submersible water bomb (Better bomb, Better Co, São Paulo, Brazil) with capacity of circulation of 60 gallons/hour. The water bomb was placed on the top of a cylinder and plastic base. The tank was sealed to prevent any kind of alteration in temperature which was measured by an accurate thermometer.

**Figure 2:**
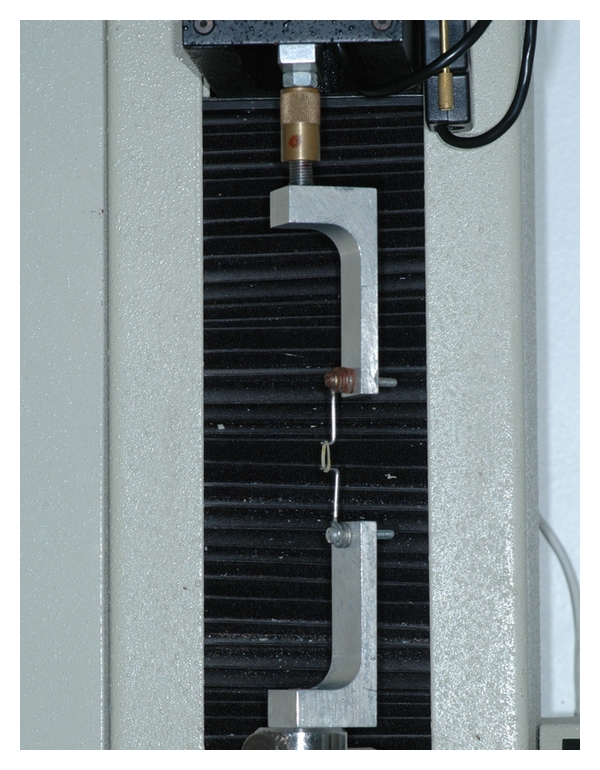
Elastic testing Emic DL 500 machine.

Elastics were mounted between stainless steel pins on an acrylic board at 30 mm distance in regular intervals of one minute. The materials remain stretched for 1, 3, 6, 12, and 24-hour period in the water tank before the force reading. Each elastic band was carefully transferred to the strain gauges cantilevers at Emic DL 500 MF testing machine (Emic Co, Sao Paulo, Brazil) (Figure 2) in the same sequence intervals, which the elastics were mounted, on the acrylic boards. The cross-head speed was 30 mm/min and the load cell capacity was 20 N (Emic Co, Sao Paulo, Brazil). Force magnitudes of the elastics when stretched at the distance of 30 mm were recorded immediately after they were removed one-by-one from the boards. The tensile readings were recorded in centi- newtons (cN) with duration of one minute for each band.

Statistical calculations were performed with 2-way analysis of variance (*P* = 0.05) to compare the effects of time intervals, different manufacturers, and Bonferroni test for multiple comparisons.

## 3. Results

Loads remaining at 1, 3, 6, 12, and 24 hours and the initial loads were determined for all types of elastics and means were recorded (Table 1). For the load relaxation from 1 hour through 24 hours, there were no consistent similarities among the AO, Tp, and Morelli elastics. The Morelli elastics maintained the greatest loads while AO maintained greater loads than Tp for 3/16, 1/4, and 5/16 elastics. The Tp showed greater loads than AO only during 0–3 hours for 3/16 elastics.

**Figure 3:**
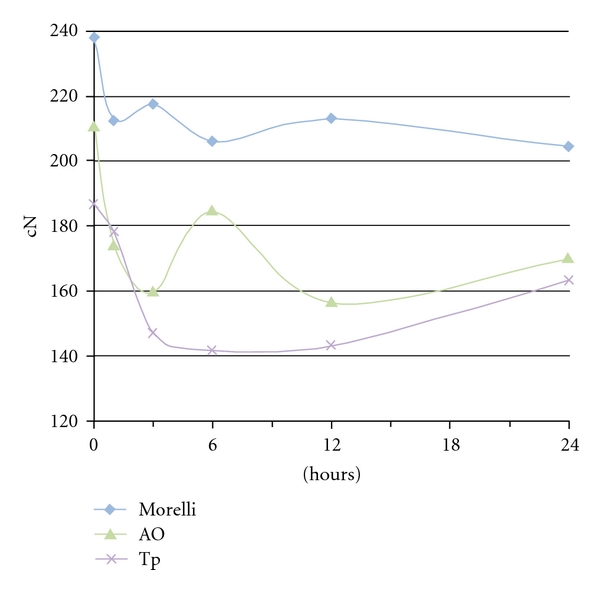
Force decay over time of 3/16 inch medium-force elastics.

**Figure 4:**
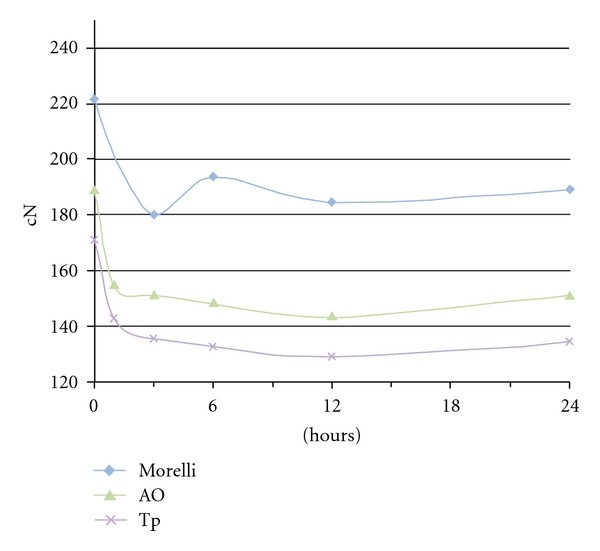
Force decay over time of 1/4 inch medium-force elastics.

**Figure 5:**
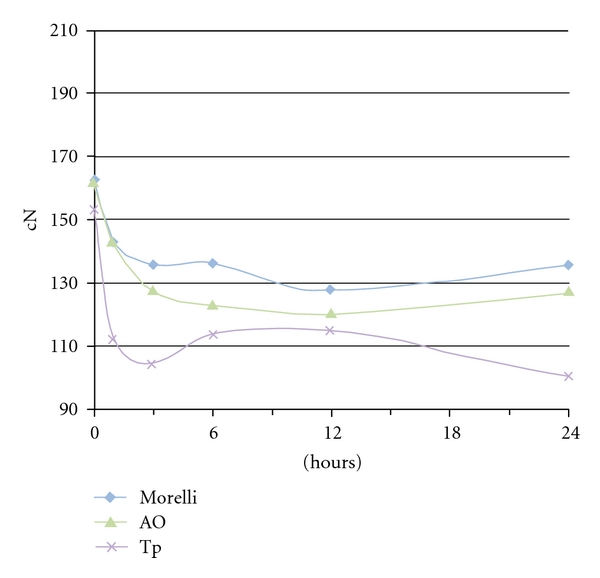
Force decay over time of 5/16 inch medium-force elastics.

The difference between the amount of force remaining after immersion in the water tank and before the stretching was calculated. After the 24-hour time interval, the differences at 3/16 elastics were 23.2 cN (Tp), 33.8 cN (Morelli), 0 cN (AO); at 1/4 elastics: 32.8 (Morelli), 35.9 cN (Tp), 38.1 cN (AO); at 5/16 elastics: 26.7 (Morelli), 34.6 cN (AO), 52.7 cN (Tp). The force decay pattern was illustrated in Figures 3, 4, and 5. A notable drop-off of forces was seen during 0–3 hours, a slight increase from 3–6 hours and more stable pattern at 6–24 hours. 

The 2-way ANOVA test was used among different groups and times. Overall, there were statistically significant differences among manufacturers (*P* < 0.0001) at different observation intervals (*P* < 0.0001). The Bonferroni test showed significant differences among all groups after multiple comparisons.

## 4. Discussion

The methods designed for this pilot could allow greater samples in a future force extension relaxation study because of the proximity between the water tank and the testing machine. Most previous studies used smaller samples as a limitation of their methods. There was limited time available to transfer each elastics from the measuring boards, together with the time required to measure force at testing machine [2, 3, 5, 6]. This method allowed greater samples because the water tank was built aside the testing machine and it could be opened to particular assess for each elastic at any time. The elastics were stretched one-by-one at measuring boards with the same regular interval used by the testing machine to assess force values. Thus, there was no difference in stretching time between the first and the fifth elastic of each group. 

 Artificial saliva was not employed because its high viscosity prevented the heated saliva circulation in tank, resulting in significant temperature variations. The method designed by a water tank with 37°C facilitated the opportunity to reveal the effect of environmental factors on the mechanical properties of the elastics.

Different extensions in wet tests were described by ranging from 20 to 40 mm, as suggested by Wang et al. [2], because these distances represent the range of elastic extensions in common clinical use and are similar to those of other studies from 20 through 50 mm [7–9]. Other authors considered values of forces provided by manufacturers as standard patterns of loads and employed extensions at 2 or 3 times the internal diameter of the elastics as references [10–12]. However, in general, the manufacturer's values could not be considered reliable once in many cases, there was great variability within samples [11]. The forces generated at 3 times diameter extension were larger than the manufacturer's values and at 2 times were smaller than the manufacturer's specified loads [12]. Thus, in this study, 30 mm of extension was employed instead of manufacturer's internal diameters loads values. 

The force extension relaxation was not evaluated in periods above 24 hours due to clinical trends [2, 3, 5]. In clinical practice, patients are usually required to discard the elastics after 1 day of use and most of them change the elastics at every meal [2]. More frequent changes could be also observed in hyperdivergent facial patients whom any kind of elastics relaxation could result in increase of vertical forces and undesirable extrusion results [13]. Even during nighttime, periods above 24 hours were not exceeded. Liu et al. suggested that the force decay was remarkably stable after 1 day of elastics usage because the structural changes caused by repeated stretching were not cumulative [4]. Furthermore, the force reduction was relatively small averaging from 2% to 6% over the second day of clinical use and remained relatively constant for a few days [2]. Other studies confirmed that after 24 hours, the force degradation could be considered nonsignificant [7, 9, 14].

There was a statistical difference in the general patterns of force-extension among the American Orthodontics, the Tp, and the Morelli elastics at all times. The results seemed to vary according to the elastic brands in each diameter group. The large initial fall-off of force of elastics subjected to immersion (Figure 2) matched findings of other studies [9, 15] and could be observed during 0–3 hours periods. The first hour fall-off averaged from 4.3 cN to 34.5 cN for 3/16 (Morelli > AO > Tp), 1/4 inch lumen (AO > Tp > Morelli), and 5/16 elastics (Morelli > AO > Tp). An elongated open downward curve characterized the Morelli and AO 3/16 elastics, Morelli 1/4 elastics, and Morelli and Tp 5/16 elastics during 3–12 hours period. The curve showed a negative degradation until 6 hours and gradual degradation from 6 to 12 hours. Further investigation is needed to determine the causes of the ascending force during 3–6 hours periods and for Tp 3/16 elastics, AO and TP 1/4 elastics, and AO 5/16 elastics from 12–24 hours. Although water immersion and temperature are significant because of inference in secondary elastics bond sites [14], perhaps a transitory hardening in material could explain the force increasing. After 12 hours, the degradation became slower, except for Tp 3/16 and 5/16 elastics. The force values followed a more straight line where the graph covers a range of forces more appropriate for most clinical applications. These comparisons of brands at different times were not evaluated in any other previously study and the differences between elastics could be explained by the characteristics of each manufacturer.

## 5. Conclusions

- For 3/16, 1/4, and 5/15 elastics, Tp showed larger force extension relaxation than AO and Morelli, respectively.
- After 6 hours, the relaxation became slower and the force values followed a more straight line over the 12–24 period where the graph should cover a range of forces more appropriate for most clinical applications.
- A guideline for in vitro mechanical analysis of latex elastics was established and allows studies under intraoral temperature.

## Figures and Tables

**Table 1:** Means and standard deviations (cN).

Diameter	Brand	Stretching periods					
		0 hour	1 hours	3 hours	6 hours	12 hours	24 hours
3/16	Morelli	238,1 (23,67)	212,5 (9,55)	217,5 (14,21)	206,1 (7,93)	213,2 (8,61)	204,3 (13,49)
	AO	174,3 (11,51)	159,7^a^ (15,09)	184,6^b^ (9,56)	156,5 (10,49)	170,0^c^ (8,12)	174,3^d^ (11,73)
	Tp	186,8 (15,81)	178,2^a^ (19,57)	146,9^b^ (9,81)	141,9 (12,52)	143,3^c^ (10,36)	163,6^d^ (15,46)

1/4	Morelli	221,7 (12,06)	201,4 (9,00)	180,0 (11,48)	193,9 (17,16)	184,7 (8,60)	188,9 (8,46)
	AO	188,9 (7,87)	154,4^e^ (4,82)	151,2 (7,17)	148,3 (4,44)	143,3 (3,24)	150,8 (5,72)
	Tp	170,4 (5,29)	142,6^e^ (8,91)	135,5 (10,55)	132,6 (8,96)	129,1 (9,97)	134,5 (4,78)

5/16	Morelli	162,2^f,l^ (10,77)	142,6^g^ (5,77)	135,5^h^ (5,91)	136,2 (5,72)	127,6^i^ (6,86)	135,5^j^ (8,07)
	AO	161,5^f,m^ (8,42)	142,6^g^ (10,16)	127,3^h^ (8,79)	123,0^n^ (9,52)	120,1^i,o^ (5,14)	126,9^j^ (5,83)
	Tp	152,9^l,m^ (4,95)	111,6 (5,86)	104,1 (5,29)	113,4^n^ (7,63)	114,8^o^ (6,38)	100,2 (3,86)

Bonferroni coefficient: same letters indicate similarity between groups (*P* > .05).
